# Improvement of Semen Quality in Holstein Bulls during Heat Stress by Dietary Supplementation of Omega-3 Fatty Acids 

**Published:** 2011-02-20

**Authors:** Hamid Gholami, Mohamad Chamani, Armin Towhidi, Mohammad Hashem Fazeli

**Affiliations:** 1 Department of Animal Science, Science and Research Branch, Islamic Azad University, Tehran, Iran; 2 Department of Animal Science, Faculty of Agricultural Science and Technology, University of Tehran, Karaj, Iran; 3 Department of Veterinary Science, Islamic Azad University of Shahrekord, Shahrekord, Iran

**Keywords:** Omega-3 Fatty Acids, Semen, Cryopreservation, *In vitro*

## Abstract

**Background:**

Long-chain polyunsaturated fatty acids (PUFAs) of the omega-3 family are important for sperm membrane integrity, sperm motility and viability. There are evidences to suggest that dietary supplementation with omega-3 fatty acids affects reproduction in men and males of different animal species. Therefore, the aim of current study was to investigate changes in the quality parameters of Holstein bull semen during heat stress and the effect of feeding a source of omega-3 fatty acids during this period.

**Materials and Methods:**

Samples were obtained from 19 Holstein bulls during the expected time of heat stress in Iran (June to September 2009). Control group (n=10) were fed a standard concentrate feed while the treatment group (n=9) had this feed top dressed with 100 g of an omega-3 enriched nutriceutical. Semen volume, sperm concentration and total sperm production were evaluated on ejaculates collected after 1, 5, 9 and 12 weeks of supplementation. Moreover, computer-assisted assessment of sperm motility, viability (eosin-nigrosin) and hypo-osmotic swelling test (HOST) were conducted.

**Results:**

Heat stress affected sperm quality parameters by weeks five and nine of the study (p<0.05). Supplementation significantly increased total motility, progressive motility, HOST-positive spermatozoa and average path velocity in the fresh semen of bulls (p<0.05).

**Conclusion:**

Dietary omega-3 supplementation improved *in vitro* quality and motility parameters of fresh semen in Holstein bulls. However, this effect was not evident in frozen-thawed semen.

## Introduction

Nutrition is a key factor in controlling fertility through its direct or indirect actions. On one hand, it directly influences the process of oocyte and spermatozoa development, ovulation and fertilization. On the other hand, the impact of nutrition on the concentration of the hormones and other metabolites can indirectly affect these processes (1).

Among nutrients, lipids have a crucial role in male fertility since they can be consumed as a source of energy and additionally they are critical components of spermatozoa membranes (2). Omega-3 fatty acids, in particular docosahexaenoic acid (DHA C22:6 ω3), are important for sperm membrane integrity, sperm motility and viability, as well as cold sensitivity (3). There are evidences to suggest that dietary supplementation with omega-3 fatty acids affects reproduction in men (4) and males of different animal species (5-9). Current evidences suggest that DHA may increase the flexibility and compressibility of the sperm tail and hence, improve the ability of the lipid bilayer to tolerate the stress of flagellar movement (10).

Extremes in climatic factors such as ambient temperature, humidity, radiation and wind influences the mammals’ environment and can deleteriously affect reproduction (11). Heat stress is the state at which mechanisms activate to maintain an animal's body thermal balance when exposed to intolerable (uncomfortable) elevated temperatures (12). Fertility is lessened during heat stress as a result of dysfunctions in reproductive processes and alterations in energy balance (13). High environmental temperatures tend to have a detrimental effect on semen production and fertility of men (14), bulls and goats (15). Exposure to hyperthermia is harmful for spermatogenesis and also decreases testosterone levels (16).

Little information is available with regard to the effect of dietary omega-3 supplementation and fertility of bulls during heat stress. Therefore, our goal was firstly to study the changes in the *in vitro* quality of Holstein bull semen during the period of heat stress and secondly, to investigate the effects of omega-3 fatty acids supplementation during this period on their reproductive performance.

## Materials and Methods

### Bulls and nutritional regimen

This experiment was conducted during the expected time of heat stress in Iran (June to September 2009). Twenty Holstein bulls housed in single pens were used for semen collection at the Semen Production Center, Karaj, Iran (35º.47' N, 50º.55' E). Bulls were divided into control (n=10) and treatment (n=10) groups balanced for bull age; however one of the bulls in the treatment group was removed from the experiment due to poor health. Control group were fed a standard concentrate feed with 15% crude protein and approximately 3 Mcal/kg metabolizable energy. Treatment group bulls had this standard feed top dressed with 100 g of a commercially available nutriceutical (Optomega 50, Optivite International Limited, Nottinghamshire, UK) which contained 25% omega-3 fatty acids (10% DHA, 6% EPA). Control and treatment bulls were fed the experimental diets for a total of 12 weeks in order to allow spermatogenesis and maturation of a new generation of spermatozoa to occur. The animals received humane care. The experiments were approved by the Research and Ethics Committee of the Science and Research Branch, Azad University, Tehran, and the experiments were performed according to international guidelines concerning the conduct of animal experimentation.

### Semen collection and evaluation

Fresh and associated frozen samples were collected after 1, 5, 9 and 12 weeks of feeding omega- 3-enriched nutriceutical, and sperm quality parameters were analyzed. Semen was collected using an artificial vagina from the bulls and immediately transferred to a 37°C water bath. Semen volume was recorded by reading from graduated tubes and sperm concentration was measured using a calibrated photometer (IMV, L’Aigle, France). The total sperm output (volume × concentration) was calculated. Subjective sperm motility assessment and viability (eosin-nigrosin) test were carried out according to Ax et al. (17) and Barth and Oko (18), respectively.

### Semen dilution and cryopreservation

Semen was diluted in a commercial diluent (Bioxcell, IMV, L’Aigle, France), pre-warmed to 37°C, to a final concentration of 40×10^6^ spermatozoa/ mL, allowing 5 minutes for the extender and semen to interact (19). Afterwards, diluted semen was packaged into 0.5 ml straws (Minitube, Germany) and for gradual cooling maintained for 4 hours at 4°C before freezing with a computer controlled freezing system (Minitube, Germany). The straws were organized on racks and placed into the freezing chamber. The computer controls the temperature inside the chamber and decreases it from 4°C to the lowest temperature chosen (-140°C) at the desired rate per minute (15°C/ min) (20, 21). After the freezing process, straws were transferred to a liquid nitrogen tank until subsequent analysis.

### Computer-assisted sperm analysis (CASA) of fresh semen

In order to perform computer-assisted assessment of sperm motility, the CASA setup (Animal Version 12.3H-CEROS, Hamilton Thorne Biosciences, Beverly, MA, USA) was pre-adjusted for bovine sperm analysis. Four microliters of diluted semen were placed in a 20 μm standard count analysis chamber (Leja, Nieuw-Vennep, The Netherlands). The loaded chamber was placed on the thermal plate of the microscope (37.5°C) for 3 minutes before analysis (22). Three randomly selected microscopic fields were scanned six times each. The mean of these 18 scans was used for statistical analysis. The following variables were analyzed: total motility (%); progressive motility (%) and average path velocity (VAP, μm/s); straight-line velocity (VSL, μm/s); and curvilinear velocity (VCL, μm/s).

### Hypo-osmotic swelling test

Plasma membrane integrity of fresh and frozen- thawed spermatozoa was assessed with the hypo-osmotic swelling test according to Revell and Mrode (23) with some modification. Hypoosmotic swelling solution (100 mOsm/kg) was prepared by dissolving 0.49 g of sodium citrate and 0.9 g fructose in 100 ml distilled water. For the HOS test, 250 μl of diluted semen was added to 1ml of the pre-warmed HOS solution and incubated at 37°C for 60 minutes. Following incubation, a 5 μl drop from each sample was transferred to a warm, clean microscope slide and covered with a 18 × 18 mm coverslip. This preparation was examined microscopically using a warm stage, × 400 magnification and phase contrast optics. Two hundred spermatozoa were counted per sample and the number of spermatozoa showing characteristic swelling of tail, an indicative of intact plasma membrane, was recorded.

### Post-thaw semen analysis

Two straws of the same batch from each treatment were thawed (37°C for 60 seconds); the semen from each straw pair was transferred into a microcentrifuge tube, and the subjective sperm motility and hypo-osmotic swelling test were performed with the same procedure described for fresh semen. The post-thaw sperm motility was evaluated using CASA, which involved placing 4μl of semen between the slide and coverslip (24). The CEROS semen analyzer, with the same setup used for fresh semen, was then employed and the same variables were analyzed.

### Calculation of temperature-humidity index (THI)

Meteorological data was collected from the weather station in closest proximity to the Artificial Insemination (AI) center. THI was calculated as follows (25):

THI=(0.8×Tdb)+[(RH100)×(Tdb-14.4)]+46.4

Where Tdb is dry bulb temperature in degrees celsius and is relative humidity (RH).

### Statistical analysis

Statistical Analysis System software (SAS Institute, version 9.1, 2002, Cary, NC, USA) was used for data analysis. Data obtained from the experimental procedure were analyzed using the Shapiro- Wilk (Proc Univariate) test to pre-determine the normality of the residues. Dependent variables that did not meet statistical premises were subjected to angular transformation [Arcsin√X +1] or logarithmic transformation [log (X + 1)]. The original or transformed data were analyzed by ANOVA for repeated measures using the Mixed Procedure of SAS in order to investigate the effects of diets, weeks of the study and the interaction between weeks and diets. Baseline values of each trait measured at the first week were used as covariate (8). Results are expressed as least square means (LSM ± SE) with a significance level of 5%.

## Results

The climatological data during the experimental period and the regarding calculated THI are shown in table 1.

Calculated THI were 70.1, 75.1, 71.6 and 70.9 during weeks 1, 5, 9 and 12 of the experiment, respectively. It can be inferred that the bulls experienced mild to moderate heat stress by weeks 5 to 9 of the study. Changes in bull semen parameters during the experimental period in both the control and omega-3 supplemented groups are shown in table 2.

Heat stress indeed affected sperm quality parameters. By week five of collection of the semen volume, sperm concentration and, as a consequence, total sperm production were higher compared to the other weeks (p>0.05). However, viability and subjective motility of fresh semen was higher during week 12 (p<0.05). The proportion of viable spermatozoa increased (p<0.05) in the ejaculates collected from omega-3-fed bulls compared to the control after 12 weeks of the feeding trial.

Changes in CASA parameters of fresh and postthaw bull semen in the control and omega-3 supplemented groups are presented in tables 3 and 4.

Dietary supplementation did not affect the subjectively assessed motility in both fresh and post-thawed semen (p>0.05). However, when motility was assessed by CASA, both total and progressive motility of fresh semen were higher after nine weeks of supplementation (p<0.01 and p<0.05, respectively) between the two groups. Similarly, the average path velocity of fresh semen was significantly higher in the omega-3-fed group in comparison with the control (p<0.05).

Semen cryopreservation was indeed found to affect sperm kinematics. The estimated losses related to the cryopreservation process were 50.41% and 53.55% for total motility and 47.93% and 49.13% for progressive motility in the control and omega-3-fed groups, respectively.

The post-thaw sperm motility parameters assessed by CASA did not significantly differ between the two groups. However, the post-thaw VAP were higher in omega-3-fed bulls after nine weeks of supplementation (p>0.05).

**Table 1:** Climatological data during the experimental period


	Ambient temperature (°C)			Relative humidity (%)			THI
	Min	Max	Mean	Min	Max	Mean

**Week 1**	17.8	33	25.4	18	49	31	70.1
**Week 5**	21	38.4	29.7	17	59	32	75.1
**Week 9**	16.0	33.8	24.9	17	59	32	75.1
**Week 12**	15.4	33.0	24.2	29	81	50	71.6


**Table 2:** Changes in bull semen parameters during experimental period in control and omega-3 supplemented groups (LSM±S.E.)


Variables (units)	Weeks of collection				Overall
	1	5	9	12	

**Semen volume (ml)**					
**Control**	5.72 ± 0.23	6.28 ± 0.23	5.68 ± 0.24	5.73 ± 0.25	5.85 ± 0.15
**Omega-3 supplemented**	5.73 ± 0.24	5.76 ± 0.24	5.07 ± 0.24	5.44 ± 0.26	5.50 ± 0.15
**Overall**	5.73 ± 0.17^a^^b^	6.02 ± 0.17a	5.37 ± 0.17^b^	5.58 ± 0.18^b^	-
**Concentration (10^6^ ml-1)**					
**Control**	1030.7 ± 34.9	1123.5 ± 34.9	1000.9± 36.4	1028.7± 38.0	1045.9 ± 26.7
**Omega-3 supplemented**	1033.5 ± 36.8	1148.3 ± 36.8	1064.7± 36.8	1065.6± 38.4	1078.0 ± 27.8
**Overall**	1032.1 ± 25.4^a^	1135.9 ± 25.4^b^	1032.8± 25.9^a^	1047.1± 27.0a	-
**Total sperm output (10^6^)**					
**Control**	5695.7 ± 290.8	6879.5 ± 290.8	5480.7± 305.6	5716.1± 321.9	5943.0 ± 197.5
**Omega-3 supplemented**	5726.3 ± 307.0	6495.8 ± 307.0	5221.8± 307.0	5663.5± 323.3	5776.8 ± 205.0
**Overall**	5711.0 ± 210.9^a^	6687.7 ± 210.9^b^	5351.3± 215.8^a^	5689.8± 227.4^a^	-
**Viability (%)**					
**Control**	64.67± 4.76	50.35± 5.71	54.26± 5.22	59.25± 4.55^A^	57.13 ± 3.04
**Omega-3 supplemented**	57.14± 4.89	53.15± 5.51	58.23± 5.67	73.64± 4.89^B^	60.54 ± 3.19
**Overall**	60.91± 3.35^a^^b^	51.75± 3.86^a^	56.24± 3.70^a^	66.44± 3.25^b^	-
**Subjective motility (%)**					
**Control**	61.73± 1.52	62.88± 1.52	60.58± 1.52	57.13± 1.57	60.58 ± 1.21
**Omega-3 supplemented**	61.58± 1.60	61.75± 1.60	60.72± 1.60	59.90± 1.66	60.99 ± 1.27
**Overall**	61.66± 1.10^a^	62.31± 1.10^a^	60.65± 1.10^a^^b^	58.52± 1.14^b^	-
**Subjective post-thaw Motility (%)**					
**Control**	37.84± 1.96	36.59± 1.96	39.76± 2.09	39.04± 2.09	38.31 ± 1.24
**Omega-3 supplemented**	37.96± 1.95	38.20± 1.85	38.76± 1.85	30.03± 1.95	38.49 ± 1.16
**Overall**	37.90± 1.38	37.40± 1.34	39.26± 1.39	39.04± 1.43	-


a, b, c Denote differences (p<0.05) in the same row. A, B,C Denote differences (p<0.05) in the same column.

Changes in the proportion of HOST-positive spermatozoa in control and omega-3 supplemented bulls are shown in table 5.

Diet enriched with omega-3 significantly increased the percentage of HOST-positive spermatozoa in fresh semen (p<0.03), wherein the two groups demonstrated the largest difference after nine weeks of nutriceutical supplementation (p<0.01). On the other hand, the effect of diet on post-thawed HOST was not significant (p=0.10), however there was an increasing tendency in the proportion of HOSTpositive spermatozoa.

**Table 3:** Changes in CASA parameters of fresh bull semen in control and omega-3 supplemented groups (LSM±SE)


Variables (units)	Weeks of collection				Overall
	1	5	9	12	

**Total motility (%)**					
**Control**	81.09 ± 1.93	84.99 ± 1.93	75.95 ± 2.04^A^	83.95 ± 2.04	81.49 ± 1.16
**Omega-3 supplemented**	78.01 ± 2.04	86.45 ± 2.04	84.90 ± 2.04^B^	88.05 ± 2.16	84.34 ± 1.21
**Overall**	79.55 ± 1.40^a^	85.72 ± 1.40^b^	80.42 ± 1.43^a^	85.98 ± 1.47^b^	-
**Progressive motility (%)**					
**Control**	58.50 ± 1.54	59.50 ± 1.54	58.15 ± 1.61^A^	62.40 ± 1.54^A^	59.64 ± 1.14
**Omega-3 supplemented**	57.91 ± 1.73	61.04 ± 1.73	64.04 ± 1.73^B^	67.14 ± 1.82^B^	62.53 ± 1.28
**Overall**	58.21 ± 1.15^a^	60.27 ± 1.15ab	61.09 ± 1.17^b^	64.77 ± 1.19^c^	-
**Average path velocity (µm/s)**					
**Control**	128.99 ± 2.49	128.99 ± 2.49	118.06 ± 2.63^A^	124.38 ± 2.63	125.11 ± 1.31
**Omega-3 supplemented**	128.21 ± 2.63	129.50 ± 2.63	126.28 ± 2.78^B^	128.61 ± 2.78	128.15 ± 1.39
**Overall**	128.60 ± 1.81^a^	129.24 ± 1.81a	122.17 ± 1.91^b^	126.50 ± 1.91^a^^b^	-
**Straight-line velocity (µm/s)**					
**Control**	102.51 ± 2.08	98.80 ± 2.08	97.29 ± 2.19	101.04 ± 2.08	99.91 ± 0.92
**Omega-3 supplemented**	102.50 ± 2.20	98.92 ± 2.20	100.83 ± 2.33	102.91 ± 2.33	101.29 ± 0.99
**Overall**	102.50 ± 1.51	98.86 ± 1.51	99.06 ± 1.60	101.97 ± 1.56	-
**Curvilinear velocity (µm/s)**					
**Control**	196.21 ± 4.62	229.17 ± 4.62	185.14 ± 4.85	198.33 ± 4.62	202.21 ± 2.73
**Omega-3 supplemented**	194.92 ± 4.87	223.82 ± 4.87	196.66 ± 5.15	202.9 ± 5.15	204.57 ± 2.92
**Overall**	195.56 ± 3.35^a^^c^	226.49 ± 3.35b	190.9 ± 3.538^a^	200.61 ± 3.46^c^	-


a, b, c Denote differences (p<0.05) in the same row. A, B,C Denote differences (p<0.05) in the same column.

**Table 4:** Changes in CASA parameters of post-thaw bull semen in control and omega-3 supplemented groups (LSM±SE)


Variables (units)	Weeks of collection				Overall
	1	5	9	12	

**Total motility (%)**					
**Control**	45.30 ± 4.48	31.56 ± 4.98	44.68 ± 4.70	40.09 ± 4.48	40.41 ± 1.99
**Omega-3 supplemented**	40.33 ± 4.72	29.11 ± 4.72	41.89 ± 4.72	45.41 ± 4.99	39.18 ± 2.02
**Overall**	42.81 ± 3.25a	30.34 ± 3.43^b^	43.28 ± 3.33^a^^c^	42.75 ± 3.35^a^^c^	-
**Progressive motility (%)**					
**Control**	31.82 ± 3.07	27.63 ± 3.07	33.44 ± 3.23	31.39 ± 3.43	31.06 ± 1.51
**Omega-3 supplemented**	29.57 ± 3.23	28.19 ± 3.24	35.27 ± 3.67	34.25 ± 3.43	31.81 ± 1.60
**Overall**	30.69 ± 2.23	27.91 ± 2.23	34.36 ± 2.45	32.82 ± 2.43	-
**Average path velocity (µm/s)**					
**Control**	111.74 ± 4.57	118.93 ± 4.57	123.56 ± 4.82	114.82 ± 4.57	117.27 ± 2.17
**Omega-3 supplemented**	111.83 ± 4.82	113.35 ±4.82	127.55 ± 4.82	122.77 ± 4.82	118.87 ± 2.25
**Overall**	111.79 ± 3.32^a^	116.14 ± 3.32^a^^b^	125.56 ± 3.40^b^	118.80 ± 3.32^a^^b^	-
**Straight-line velocity (µm/s)**					
**Control**	91.12 ± 3.83	105.50 ± 3.83	103.42 ± 4.04	95.44 ± 3.83	98.87 ± 1.92
**Omega-3 supplemented**	92.03 ± 4.04	96.77 ± 4.04	107.60 ± 4.04	103.35 ± 4.04	99.94 ± 2.00
**Overall**	91.57 ± 2.78^a^	101.13 ± 2.78^b^	105.51 ± 2.86^b^	99.39 ± 2.78^b^	-
**Curvilinear velocity (µm/s)**					
**Control**	188.28 ± 7.97	183.21 ± 7.97	189.73 ± 8.34	176.16 ± 7.97	184.34 ± 2.77
**Omega-3 supplemented**	186.60 ± 8.41	172.43 ± 8.41	193.52 ± 8.41	187.48 ± 8.41	185.01 ± 2.85
**Overall**	187.44 ± 5.79	177.82 ± 5.79	191.62 ± 5.92	181.82 ± 5.79	-


a, b, c Denote differences (p<0.05) in the same row.

**Table 5:** Changes in proportion of HOST-positive spermatozoa in control and omega-3 supplemented bulls (LSM±SE)


Parameters (unit)	Weeks of collection				Overall
	1	5	9	12	

**Fresh HOST (%)**					
**Control**	55.55 ± 1.92	57.24 ± 1.92	51.80 ± 2.02^A^	64.58 ± 2.03	57.29 ± 1.08^A^
**Omega-3 supplemented**	55.34 ± 2.03	60.34 ± 2.03	59.73 ± 2.03^B^	69.46 ± 2.15	61.22 ± 1.12^B^
**Overall**	55.44 ± 1.40^a^	58.79 ± 1.40^a^	55.76 ± 1.43^a^	67.02 ± 1.48^b^	-
**Post-thaw HOST (%)**					
**Control**	22.57 ± 2.18	23.79 ± 2.18	31.85 ± 2.18	33.96 ± 2.18	28.04 ± 1.24
**Omega-3 supplemented**	23.62 ± 2.18	24.93 ± 2.18	31.81 ± 2.18	37.52 ± 2.31	29.47 ± 1.25
**Overall**	23.09 ± 1.54^a^	24.36 ± 1.54^a^	31.83 ± 1.54^b^	35.74 ± 1.58^b^	-


a, b Denote differences (p<0.05) in the same row A, B Denote differences (p< 0.05) in the same column

## Discussion

It can be inferred from the meteorological data and calculated THIs that by week five of the experiment, the highest heat stress had occurred. When the THI exceeds 72, cattle are affected adversely (26). Subsequently, there was a significant decreasing tendency in semen volume, sperm concentration and total sperm production until week nine of the study. A testicular temperature that is below body temperature is known to be essential for the production of fertile spermatozoa (27). Higher ambient temperature may result in increased testicular temperatures and thus decrease semen quality. Spermatogenesis and epididymal maturation in bulls takes about 65 days (28). The results for ejaculate volume were in accordance with Meyerhoeffer et al. (29). In their experiment in which AI bulls were exposed to heat, the authors had observed a decrease in ejaculate volume during the first six weeks of heat stress. Similar to the present results, Meyerhoeffer et al. (29) have reported a decrease of the percentage of motile sperm two weeks after heat exposure was noted. They assumed that heat stress might not affect epididymal sperm while an impact during spermatogenesis exists.

In this study feeding an omega-3 enriched nutriceutical improved the motion characteristics of fresh sperm assessed by CASA. Total motility, progressive motility and average path velocity of nutriceutical-fed bulls were significantly higher than the control group after nine weeks of the feeding trial. Our results were consistent with those of Conquer et al. (4) in humans, Rooke et al. (8) in boars, Dolatpanah et al. (30) in goats and Towhidi et al. (31) in sheep who reported significant correlations between dietary omega-3 supplementation and the number of motile spermatozoa. However, in contrast, studies in boars (7), stallions (9) and turkeys (6) found no evidence of a positive effect of dietary omega-3 fatty acid supplementations on fresh semen motility. DHA is the major long chain polyunsaturated fatty acids (PUFAs) found in the phospholipids of the spermatozoa in mammals (32). Conner et al. (33) have observed that the majority of DHA in monkey sperm is located in the tail, while phospholipids in the head region contain very low amounts of DHA. In their study, it was estimated that, surprisingly, the tail contained 99% of the total DHA of spermatozoa. Moreover, in a recent study Mourvaki et al. (34) reported that dietary supplementation of flaxseed increased the proportions of PUFAn-3, mainly DHA in rabbit sperm. They observed that the tail was the region mostly affected by the flaxseed treatment and a major increase (from 3.3% to 19.3% of total fatty acids) was detected in the DHA content of the tail. To a certain extent, it can be inferred that the high content of this long chain polyunsaturate in the sperm tail may contribute to the flagellar action and bending movement required for motility. The DHA acyl-chain in phospholipids bilayers is highly flexible and can rapidly convert between an extended and a looped conformation. This confers springy quality to the membrane, allowing it to accommodate and recover from compressive forces in the lateral plane of the bilayer. Membranes with a high content of DHA in their phospholipids are, therefore, distinguished by high levels of flexibility, compressibility, deformability, and elasticity (10). Hence, it can be suggested that DHA may have an important role in the physiological and molecular mechanisms controlling the spermatozoa membrane fluidity.

Dietary supplementation did not enhance semen volume, sperm concentration and total sperm production which are in agreement with the results of Adeel et al. (35) in buffalo, Cerolini et al. (36) in chickens and Zaniboni et al. (6) in turkeys. However, feeding a source of omega-3 fatty acids leads to a higher sperm concentration and total production in boars (8) and stallions (9). Different results have reported that the effects of DHA on sperm concentration and total production might be related to the proportion of DHA and docosapantaenoic acid (DPA C22: 5n-6) in the semen of boars and stallions. The semen of boars contains very high levels of DPA and the semen lipid profile of stallions is similar to that of the boar (8,9). Quite the opposite, DHA is the predominant PUFA in bull spermatozoa whereas DPA is extremely low (37). We observed an increasing tendency in the percentage of swelling with the hypo-osmotic test throughout the experiment. It can be suggested that feeding omega-3 enriched nutriceutical may increase the incorporation of DHA in to the principle piece, facilitating sperm membrane stability against hypoosmotic solution. On the other hand, increases in the number of viable cells is again similar to the results obtained in boars (8) and turkeys (6), but in contrast to the results reported in stallions (9). Results may vary according to the method applied to assess sperm plasmalemma. Brinsko et al. (9) used flow cytometry for evaluating viable cells but the others assessed viability by means of eosinnigrosin. Despite the suggested structural role of PUFA in plasmalemma of sperm, DHA incorporation into spermatozoa might enhance a series of actions that lead to prevention of early apoptosis as it has been seen in cultured neurons and retinal photoreceptors (38). This may promote higher numbers of viable spermatozoa in the ejaculate.

## Conclusion

It can be concluded that heat stress has detrimentally decreased sperm quality parameters in Holstein bulls. Dietary omega-3 supplementation or its precursors, improve *in vitro* quality and motility parameters of fresh semen assessed by CASA in Holstein bulls. However, this effect was not obvious in frozen-thawed semen.

## References

[1] Wathes DC, Abayasekara DR, Aitken RJ (2007). Polyunsaturated fatty acids in male and female reproduction. Biol Reprod.

[2] Santos JE, Bilby TR, Thatcher WW, Staples CR, Silvestre FT (2008). Long chain fatty acids of diet as factors influencing reproduction in cattle. Reprod Domest Anim.

[3] Robinson JJ, Ashworth CJ, Rooke JA, Mitchell LM, McEvoy TG (2006). Nutrition and fertility in ruminant livestock. Anim Feed Sci Technol.

[4] Conquer JA, Martin JB, Tummon I, Watson L, Tekpetey F (2000). Effect of DHA supplementation on DHA status and sperm motility in asthenozoospermic males. Lipids.

[5] Kelso KA, Cerolini S, Noble RC, Sparks NH, Speake BK (1997). The effects of dietary supplementation with docosahexaenoic acid on the phospholipid fatty acid composition of avian spermatozoa. Comp Biochem Physiol B Biochem Mol Biol.

[6] Zaniboni L, Rizzi R, Cerolini S (2006). Combined effect of DHA and alpha-tocopherol enrichment on sperm quality and fertility in the turkey. Theriogenology.

[7] Paulenz H, Taugbøl O, Kommisrud E, Grevle IS (1999). Effect of dietary supplementation with cod liver oil on cold shock and freezability of boar semen. Reprod Domest Anim.

[8] Rooke JA, Shao CC, Speake BK (2001). Effects of feeding tuna oil on the lipid composition of pig spermatozoa and in vitro characteristics of semen. Reproduction.

[9] Brinsko SP, Varner DD, Love CC, Blanchard TL, Day BC, Wilson ME (2005). Effect of feeding a DHA-enriched nutriceutical on the quality of fresh, cooled and frozen stallion semen. Theriogenology.

[10] Speake BK, Surai PF, Rooke JA, DeVriese S, Christophe AB (2003). Regulation of avian and
mammalian sperm production by dietaryfatty acids. Male fertility and lipid
metabolism.

[11] Gwazdauskas FC (1984). Effects of climate on reproduction in cattle. J Dairy Sci.

[12] Marai IFM, Haeeb AAM (2010). Buffalo’s biological functions as affected by heat stress- A review. Livestock Science.

[13] De Rensis F, Scaramuzzi RJ (2003). Heat stress and seasonal effects on reproduction in the dairy cow-a review. Theriogenology.

[14] Sinclair S (2000). Male Infertility: Nutritional and environmental considerations. Altern Med Rev.

[15] Murugaiyah M, Lokeshan RR (1992). Changes in the semen characteristics of
Kambing Katjan crossbreed buck under hot and humid
environmental temperatures. Recent advances in goat production.

[16] Murray MT (1997). Male infertility: a growing concern. Am J Natural Med.

[17] Ax RL, Dally MA, Lenz RW, Love CC, Varner DD, Hafez B, Hafez B, Hafez ESE (2000). Semen Evaluation. Reproduction in farm animals.

[18] Barth AD, Oko RJ (1989). Abnormal morphology of bovine spermatozoa.

[19] Barati F, Khaksary Mahabadi M, Mohammadi Gh (2009). Effects of two different diluents on the buffalo epididymal sperm cryopreservation. Yakhteh Medical Journal.

[20] Sharafi M, Forouzanfar M, Hosseini SM, Hajian M, Ostadhosseini S, Hosseini L (2009). In Vitro comparison of soybean lecithin based-extender with commercially available extender for ram semen cryopreservation. International Journal pof Fertility and Sterility(JFS).

[21] Baracaldo MI, Barth AD, Bertrand W, IVIS Steps for freezing
bovine semen: from semen collection to the liquid nitrogen
tank. IVIS reviews in veterinary medicine.

[22] Goovaerts IG, Hoflack GG, Van Soom A, Dewulf J, Nichi M, Kruif A (2006). Evaluation of epididymal semen quality using the Hamilton-Thorne analyser indicates variation between the two caudae epididymides of the same bull. Theriogenology.

[23] Revell SG, Mrode RA (1994). An osmotic resistance test for bovine semen. Anim Reprod Sci.

[24] Celeghini ECC, de Arruda RP, de Andrade AFC, Nascimento J, Raphael CF, Rodrigues PHM (2008). Effects that bovine sperm cryopreservation using two different extenders has on sperm membranes and chromatin. Anim Reprod Sci.

[25] Mader TL, Davis MS, Brown-Brandl T (2006). Environmental factors influencing heat stress in feedlot cattle. J Anim Sci.

[26] Armstrong DV (1994). Heat Stress interaction with shade and cooling. J Dairy Sci.

[27] Waites GMH, Johnson AD (1970). Temperature regulation and the testis. The Testis.vol1.

[28] Saunders PT (2003). Germ cell-somatic cell interactions during spermatogenesis. Reprod Suppl.

[29] Meyerhoeffer DC, Wettemann RP, Coleman SW, Wells ME (1985). Reproductive criteria of beef bulls during and after exposure to increased ambient temperature. J Anim Sci.

[30] Dolatpanah MB, Towhidi A, Farshad A, Rashidi A, Rezayazdi A (2008). Effect of fish oil on semen quality of goats. Asian Aust Anim Sci.

[31] Towhidi A, Samadian F, Rezayazdi K, Rostami F, Ghaziani F (2008). Feeding n-3 fatty acid source improves semen quality
by increasing n3/n6 fatty acids ratio in sheep. Book of abstracts
of 10th World Conference on Animal Production.

[32] Poulos A, Sharp P, Johnson D, White I, Fellenberg A (1986). The occurrence of polyenoic fatty acids with greater than 22 carbon atoms in mammalian spermatozoa. Biochem J.

[33] Connor WE, Lin DS, Wolf DP, Alexander M (1998). Uneven distribution of desmosterol and docosahexaenoic acid in heads and tails of monkey sperm. J Lipid Res.

[34] Mourvaki E, Cardinali R, Dal Bosco A, Corazzi L, Castellini C (2010). Effects of flaxseed dietary supplementation on sperm quality and lipid composition of sperm subfractions and prostatic granules in rabbit. Theriogenology.

[35] Adeel M, Ijaz A, Aleem M, Rehman H, Yousaf MS, Jabbar MA (2009). Improvement of liquid and frozen-thawed semen quality of Nili-Ravi buffalo bulls (Bubalus bubalis) through supplementation of fat. Theriogenology.

[36] Cerolini S, Zaniboni L, Maldjian A, Gliozzi T (2006). Effect of docosahexaenoic acid and α-tocopherol enrichment in chicken sperm on semen quality, sperm lipid composition and susceptibility to peroxidation. Theriogenology.

[37] Kelso KA, Redpath A, Noble RC, Speake BK (1997). Lipid and antioxidant changes in spermatozoa and seminal plasma throughout the reproductive period of bulls. J Reprod Fertil.

[38] Neuringer M, Anderson GJ, Conner WE (1988). The essentiality of n-3 fatty acids for the development and function of the retina and brain. Annu Rev Nutr.

